# Does the Immunocompetent Status of Cancer Patients Have an Impact on Therapeutic DC Vaccination Strategies?

**DOI:** 10.3390/vaccines6040079

**Published:** 2018-11-23

**Authors:** Silvia Martin Lluesma, Michele Graciotti, Cheryl Lai-Lai Chiang, Lana E. Kandalaft

**Affiliations:** 1 Center of Experimental Therapeutics, Ludwig Center for Cancer Research, Department of Oncology, University of Lausanne, Lausanne 1011, Switzerland; Silvia.Martin-Lluesma@chuv.ch; 2 Vaccine development laboratory, Ludwig Center for Cancer Research, Lausanne 1011, Switzerland; Michele.Graciotti@chuv.ch (M.G.); Lai-Lai-Cheryl.Chiang@chuv.ch (C.L.-L.C.)

**Keywords:** DC vaccine, immunotherapy, cancer

## Abstract

Although different types of therapeutic vaccines against established cancerous lesions in various indications have been developed since the 1990s, their clinical benefit is still very limited. This observed lack of effectiveness in cancer eradication may be partially due to the often deficient immunocompetent status of cancer patients, which may facilitate tumor development by different mechanisms, including immune evasion. The most frequently used cellular vehicle in clinical trials are dendritic cells (DCs), thanks to their crucial role in initiating and directing immune responses. Viable vaccination options using DCs are available, with a positive toxicity profile. For these reasons, despite their limited therapeutic outcomes, DC vaccination is currently considered an additional immunotherapeutic option that still needs to be further explored. In this review, we propose potential actions aimed at improving DC vaccine efficacy by counteracting the detrimental mechanisms recognized to date and implicated in establishing a poor immunocompetent status in cancer patients.

## 1. Therapeutic DC Vaccines in Cancer Patients: Context and Current Situation

Dendritic cells (DCs) are frequently used in clinical trials as they are considered an ideal vehicle for antigen delivery [1]. Discovered in 1973 by Ralph Steinman and Zanvil A. Cohn [2], DCs are sentinels of the immune system that initiate and direct immune responses [3]. Dendritic cells are activated by the sensing of danger in the form of pathogen or damage-associated molecular patterns (PAMPs or DAMPS, respectively), which they uptake, process, and present as antigenic peptides to naïve T-cells in peripheral tissues. Dendritic cells therefore constitute the most important antigen-presenting cell (APC) population for activating antitumor T-cell responses; they play a critical role in the interface between innate and adaptive immunity [4]. The aim of vaccination is to increase tumor-associated antigen (TAA) presentation to the immune system, and hence increase the activation of tumor-specific T and B cells. Human DCs can be generated ex vivo from CD34^+^ hematopoietic progenitors or from peripheral blood-derived monocytes [5]. Several vaccination procedures are currently available and have been successfully tested in the clinic [6]. Most frequently, DCs are loaded ex vivo with different antigens, such as whole tumor lysate, peptides, proteins, or genetic material of the desired antigen (e.g., transfected/electroporated DNA, RNA or transduced virus), prior to reinfusion into the patient [7]. Alternatively, DCs can be given alone after cytotoxic therapy (such as chemo- or radiotherapy), whereby increased antigen availability could be induced in vivo. One important observation is that DC vaccination as a monotherapy is generally considered safe as grade 3 or 4 treatment-related toxicities are rare [8,9]. These data were confirmed by several phase III trials where DC vaccination had been compared with placebo [10,11,12,13]. Furthermore, DC vaccination is associated with preserved quality of life of cancer patients [14].

Currently, DC vaccines are being revisited as a potential tool in the immunotherapeutic arsenal [15]. Interestingly, DC therapy has been shown to produce a similar objective response rate (ORR) than standard therapy in melanoma, prostate cancer, malignant glioma, and renal cell carcinoma according to a meta-analysis of all the published DC vaccine clinical trials [16]:

1. In melanoma, DC therapy had 8.5% ORR, similar to dacarbazine (standard of care), or ipilimumab (5–15%)
2. In prostate cancer patients, ORR was 7.1% after DC vaccination, compared to 10% with conventional chemotherapeutic drugs
3. In patients with malignant glioma, ORR after DC therapy was 15.6%
4. In advanced renal cell cancer (RCC), ORR was 11.5%

Of note, an increase of at least 20% in overall survival (OS) has been documented in most studies using DC therapies [16]. However, survival was not the main endpoint in many of these studies as they were early phase. Nevertheless, clinical efficacy for DC vaccination is still far from optimal and this might probably be associated with their inability to elicit a rapid and strong T cell response. Consequently, this has generated a great deal of criticism towards therapeutic vaccination [17], including DC vaccination. The lack of therapeutic vaccination efficacy is probably associated with a dampened immunocompetent status in cancer patients, and the presence of immunosuppressive mechanisms in different indications that allow tumor development. This could be due to some common phenomena shared by all tumor types, independent of the specific characteristics of each one. These potential obstacles could be overcome by implementing procedures to improve patients’ immunocompetent status and optimizing vaccination design, although differential responses are probably expected in different genetic backgrounds, requiring then a tailored approach (further discussed in Section 3.3.1.2). Therefore, novel strategies that increase DC vaccination efficacy while maintaining its safety profile are warranted.

## 2. Determinant Factors Impinging on the Efficacy of Therapeutic Vaccination

In a recent clinical trial in melanoma patients, it was observed that tumor-specific immunologic response rates obtained after DC vaccination in the adjuvant setting were approximately two to three times higher than in the metastatic setting [18]. This suggested that using DC-based immunotherapy earlier in the course of the disease when tumor burden is still minimal might positively influence the vaccination efficacy. In a meta-analysis of 54 trials using DC vaccination on 967 melanoma patients, the observed ORR was similar between stage III and IV diseases but the clinical response was statistically different between the two groups (*P* = 0.03). Furthermore, progressing disease (PD) cases were significantly different between stages II (18.8%) and IV (52.6%), and between stages III (23.1%) and IV (both *P* = 0.0001) [19]. These observations supported the notion that reduced immunocompetence was associated with tumor progression.

**Figure 1:**
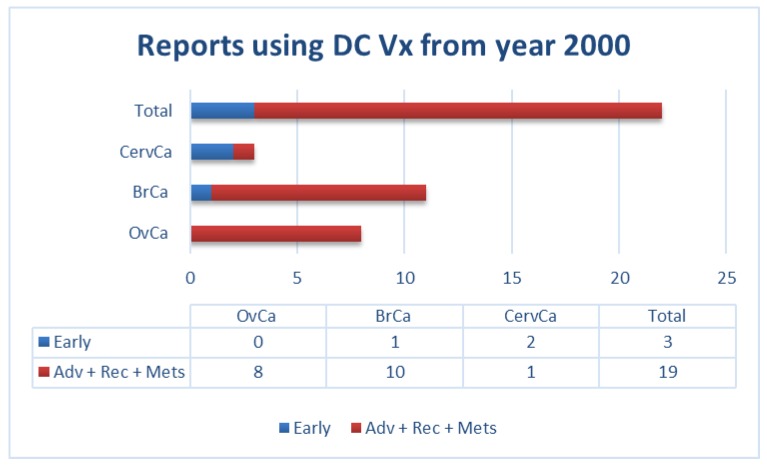
Number of reports in literature (source: PubMed) using dendritic cell (DC) vaccination in patients with the indicated gynecological and breast cancers (not exhaustive; according to data summarized in Supplementary Materials Table S1). Adv: advanced; BrCa: breast cancer; CervCa: cervical cancer; Mets: metastatic; OvCa: ovarian cancer; Rec: recurrent.

Similarly, our own literature review of clinical trials using DCs as a vehicle (for all types of antigenic sources) in gynecological and breast cancers from the year 2000 to date, showed that most of these trials were performed in patients with advanced diseases. This factor could potentially contribute to the limited success of vaccination in these patients (Figure 1).

In the studies we have summarized above (Figure 1 and Supplementary Materials Table S1), patients with gynecological cancers (i.e., ovarian, cervical) or breast cancer were vaccinated with: i) native DCs [20]; ii) DCs loaded either with antigen-specific peptides [21,22,23,24,25,26,27,28], a viral protein [29,30], or with autologous tumor lysate [31,32,33,34,35,36,37,38,39]; or iii) DCs fused with autologous tumor cells [40]. In some cases, DCs were activated with interferon (IFN)-γ alone [32], lipopolysaccharides (LPS), and IFN-γ [34,35,36,39], or IFN-γ in combination with other compounds [26]. In other cases, tumor necrosis factor (TNF)-α was used either alone [28,31,38] or in combination with either interleukin (IL)-1β [37], or IL-1β plus IL-6 and prostaglandin-E2 (PGE2) [29,30,33]. In numerous early studies, no DC maturation stimulus was used [20,21,22,23,24,25,40]. As indicated in Figure 1, most patients in these studies had advanced metastatic or recurrent diseases. In most of these studies, patients presented a somewhat increased immunogenicity upon vaccination, including peptide-specific CD8^+^ T-cells [21,22,24,26,28,29,30,36,39,40], IFN-α production by injected DCs [20], tumor antigen-specific lymphoproliferative response [31], increased IFN-γ secreting cells [32,33,38], increased frequency of CD4^+^ CD25 high T cells [25], potent Th1 polarization [34,35], or delayed-type hypersensitivity (DTH) responses [29,33]. However, the observed increased immunogenicity only rarely correlated with some clinical benefits such as temporary tumor regression [20,40], partial responses [21,27,36], disease stabilization [22,24,27,31,32,36,40], or extended progression-free survival (PFS)/time to recurrence (TTR) [26,29,33,34,37,38].

As therapeutic vaccination has so far shown limited efficacy in advanced diseases, this supports the notion of vaccinating cancer patients with early-stage diseases or after debulking procedures whenever possible. It is also important to elucidate the mechanisms that contribute to a reduced immunocompetent status in patients with advanced diseases. This would help in designing appropriate vaccination strategies for boosting the patient’s immune system and improving overall therapeutic efficacy in the advanced disease setting as well.

### 2.1. Tolerance Mechanisms Exerted by Tumors and Their Microevironment to Evade Immune Recognition

Tumor progression is generally associated with an immunosuppressive tumor microenvironment (TME), as tumor cells develop tolerance mechanisms to inhibit relevant T cell repertoires leading to immune escape [41]. Tumors have evolved different evasion mechanisms including: i) downregulation of major histocompatibility complex (MHC) Class I via the inhibition of NLRC5, which is a key transcription cofactor, to avoid T cell recognition [42,43]; ii) expression of surface molecules for T cell suppression, such as programmed-death ligand 1 (PD-L1) [44,45], galectin-9 [46,47], and galectin-3 [48]; iii) production of immunosuppressive molecules (e.g., indoleamine 2,3-dioxygenase [IDO] [49,50]), and cytokines (e.g., IL-6 [51], IL-10, and transforming growth factor [TGF]-β); iv) promoting T regulatory (Treg) cell proliferation [52,53,54,55]); and v) an increased presence of immature myeloid cells [56].

Concerning gynecological and breast cancers, in cervical carcinoma, TNF-α (a potent activator of Langerhans cells [LCs]), was constitutively expressed by basal keratinocytes in the normal cervix in an early study. However, it was absent in the majority (32 out of 41) of low- and high-grade cervical intraepithelial neoplasia (CIN) [57]. In addition, IL-10 was upregulated in 20 out of 41 CIN lesions and was absent in normal epithelial. Moreover, LCs in the CIN lesions did not express adhesion/costimulation molecules for T cell activation, suggesting that the aberrant expressions of TNF-α and IL-10 played a role in inhibiting the anti-tumor functions of LCs.

In ovarian carcinoma, the ascites often constitute a highly immunosuppressive network of immune cells, cytokines, chemokines, tumor cells, and non-immune cells (e.g., fibroblasts, adipocytes, and mesothelial cells) that help to shape the immune response. For example, ascites-derived vascular endothelial growth factor (VEGF) [58,59], IL-6, IL-10, TGF-β, and arachidonic acid played a prominent role in the polarization of immunosuppressive tumor-associated macrophages (TAMs) in ovarian cancer ascites [60,61,62,63]. Such polarized TAMs participate in numerous pathological processes including assisting tumor cell invasion, angiogenesis, and metastasis [64]. Moreover, the presence of the CD163 activation marker on TAMs in ovarian cancer ascites strongly correlates with early relapse of serous ovarian carcinoma after first-line therapy [60].

Similarly, the TME of solid tumors such as breast carcinoma is composed of stromal fibroblasts, vasculature, inflammatory immune cells, and extracellular matrix that interact with the tumor cells via cytokines, growth factors, proteases, and other molecules. These interactions also regulate the anti-tumor responses within the TME, ensuring the survival of the tumor. In breast carcinoma, the tumor cells were shown to produce TGF-β and IDO. They also repressed the expression of activating receptors such as natural killer (NK)p30 and NKG2D on NK cells, hence reducing the ability of NK cells to directly lyse tumors [65].

Treg cells are frequently recruited into the TME, and this phenomenon has been well-characterised in different cancer types. Their increased presence has been correlated with advanced cancer stage in hepatocellular carcinoma [66] and reduced survival in ovarian cancer patients [54]. It has been shown that tumor cells and DCs in the TME produced TGF-β [67,68] and expressed B7-H1 [69] to support the differentiation of these Treg cells. Consequently, the Treg cells could inhibit effector T-cells through the secretions of IL-10 and TGF-β, and via cell–cell contact with the latter [70]. Myeloid-derived suppressor cells (MDSCs) could also be recruited into the TME and release T cell inhibitors such as arginase and nitric oxide synthase [71]. Myeloid-derived suppressor cells are capable of suppressing T cell and NK cell activities [72], as well as enhancing cancer progression and metastasis [73]. Finally, it has been shown that the critical soluble mediators of type-1 immune effector cells, IFNγ and TNFα, could synergize in the induction of COX-2 which is a key enzyme in PGE2 synthesis. As PGE2 could participate in T cell immunosuppression, we could potentially prevent that with COX-2 blockade [74].

### 2.2. Reduced DC Fitness in Cancer Patients vs. Healthy Subjects

#### 2.2.1. Peripheral Blood DC Defects in Cancer Patients

Several studies have highlighted quantitative and functional impairments of blood-circulating DCs in many different types of cancer [75], including melanoma [76], colorectal cancer (CRC) [77,78], prostate adenocarcinoma [79], head and neck cancer (HNCSC) [80], breast cancer [81,82], pancreatic cancer [83], and hepatocellular carcinoma [84]. Despite using different methodologies and patient cohorts leading to few minor discrepancies, these studies have collectively demonstrated that the DC cell compartment is defective in cancer patients, compared to healthy subjects.

In particular, a statistically significant and marked reduction in the total number of DCs circulating in the peripheral blood (PB-DCs) has been observed in cells belonging to either the myeloid lineage (e.g., in HNCSC [80], breast cancer [81], and hepatocellular carcinoma [84]), the lymphoid lineage (e.g., localized prostate cancer (close to statistical significance, *p* = 0.055) [79], and stage III melanoma [76]), or both (e.g., stage IV melanoma [76], and advanced metastatic prostate cancer [79]).

Importantly, in studies analyzing different disease stages, the observed decrease in PB-DCs correlated well with cancer progression, with an attenuated or undetectable effect at early stages (I-II), but a significant and more pronounced decline in patients presenting a more advanced disease (stage III-IV) [76,85]. Furthermore, analyses of PB-DCs in patients before and after tumor surgical removal showed a reverse trend towards normalization in terms of DC numbers [80,81,86,87,88], highlighting the fact that the observed numerical deficiencies were tumor-induced and indeed reversible upon tumor debulking.

In addition to this, important DC functional impairments have also been reported in several types of cancer. Della Bella and colleagues for example reported that while the maturation response of PB-DCs to LPS did not differ between healthy subjects and patients, the percentage of PB-DCs expressing IL-12 was significantly lower in the latter [81]. An analogous decrease in IFN-α production by tissue-resident plasmacytoid DCs (pDCs) in cancer patients was reported by Hartmann et al. in the context of HNCSC [89]. Importantly, in three other studies the PB-DCs stimulatory capacity, as measured by standard mixed leukocyte reactions (MLRs), was significantly impaired in PB-DCs isolated from breast [82], pancreatic [88], and colorectal [87] cancer patients compared to healthy subjects. In particular, in the latter study this aspect correlated well with a significantly lower expression of human leukocyte antigen (HLA)-DR, CD83, CD86, and mannose receptor, all indicative of a more DC immature state [87], and thus potentially more tolerogenic [90,91].

Some studies have also given a few interesting mechanistic insights on how these deficiencies may emerge, a notion that is obviously particularly valuable in the attempt to advance current anticancer immunotherapies. Since the earlier studies, a crucial role has been attributed to soluble factors released by tumor cells, particularly to VEGF [92]. This signalling molecule is in fact not only essential to ensure neo-angiogenesis and subsequent tumor growth and progression [93], but it has also important, proven immunoregulatory effects, such as: i) hampering DC development from CD34^+^ cell precursors [94], ii) impairment of DC maturation [95], and iii) induction of reduced in vitro T cell stimulation capability by DCs [96]. Interestingly, in line with these data, it has been shown that VEGF serum levels in CRC patients inversely correlates with PB-DC circulating levels [87], hence reinforcing the view that VEGF plays a dual role in tumor progression, contributing to both tumor angiogenesis and immune escape.

IL-6 and IL-10 are two other growth factors released by cancer cells able to induce immune system deficiencies. Beckebaum and colleagues showed in patients with hepatocellular carcinoma that in vitro exposure of peripheral blood mononuclear cells (PBMCs) to IL-10 led to a significant decrease in cell occurrence, and decreased expression of co-stimulatory molecules, in both myeloid and pDC subsets [97]. Importantly, the same study also evidenced a clear correlation between increased IL-10 serum levels, and numerical and mature phenotype deficiencies in patients’ PB-DCs [97]. Regarding IL-6, this cytokine has been shown to arrest the development of DCs from CD34^+^ cell precursors [98], whereas suppressing DC maturation [99], and T cell activation capability [99,100].

In the context of ovarian cancer, no previous work investigated the phenotype and occurrence of blood circulating DCs in cancer patients to our knowledge; however, several studies reported important dysfunctions of DCs present in the ovarian tumor microenvironment (TME) [101]. The ovarian cancer TME is particularly immunosuppressive with high levels of immunoregulatory mediators such as PGE2, IL-10, VEGF and others, all correlating with tumor progression and poor prognosis [102]. These different stimuli are able to shape and induce tumor-infiltrating DCs (TIDCs) towards an immunosuppressive and regulatory phenotype [103,104,105]. Work done in mouse models that closely recapitulate human ovarian cancer interestingly showed that malignant progression was triggered by tumor-induced phenotypical changes in TIDCs, an effect that was reversed upon DC depletion [106]. Beneficial effects of DC depletion towards slowing down ovarian tumor progression were also reported in another previous study [107]. These results have been linked to both the tolerogenic role played by TIDCs in supporting immune surveillance escape [106], and to their ability to promote angiogenesis and maintainance of tumor vasculature [107,108], thus suggesting novel opportunities for therapeutic intervention, specifically against ovarian cancer (see also further Section 3.3.2.4).

In conclusion, many studies reported important dysfunctions in the number and immunogenic phenotype of blood circulating DCs in cancer patients. While no data has been reported so far in the case of ovarian cancer, the evidence of major dysfunction in TIDCs and the known ability of factors present in the ovarian TME (such as VEGF) to systemically inhibit DC generation from hematopoetic cell precursors [95] suggests that impairments similar to those found in other cancer types should also be present in ovarian cancer patients. Future analyses should address this point to further advance vaccination therapies against ovarian cancer.

#### 2.2.2. Defects of Monocyte-Derived DCs in Cancer Patients

In the case of monocyte-derived DCs (mo-DCs) cultured ex-vivo for therapeutic vaccination, important defects have been reported when compared to mo-DCs derived from healthy subjects, although with some discrepancies among different studies. A comparison between mature mo-DCs from CRC and non-small cell lung cancer (NSCLC) patients, and healthy subjects showed, for example that: i) mo-DCs from healthy donors had a more mature phenotype (higher CD83 and CCR7; lower CD14) than DCs from cancer patients; ii) mo-DCs from CRC patients had a more mature phenotype (higher CD83 and CCR7) than mo-DCs from NSCLC patients; and iii) maturation status of DCs correlated positively with the patients’ clinical status [109]. Maybe not surprisingly, the authors in this study also observed that in clinical responders, DCs generated from monocytes isolated after the 4th vaccine had a more mature phenotype (higher CD83 and CCR7) than DCs obtained from monocytes isolated prior to treatment [109], thus suggesting that DC vaccination also had a positive influence on the immunocompetence status of responders. Similarly, a second study reported deficiencies in the maturation status, immunogenic cytokine profile, and T-cell stimulation capacity of mo-DCs derived from CRC patients compared to healthy controls, which inversely correlated with disease progression, and which were partially restored to normal levels after surgery [110].

Concerning gynecological and breast cancers, another study compared mo-DCs phenotypes between breast cancer patients and healthy donors using three different maturation stimuli [111]. In this work, the yield of mo-DCs from breast cancer patients was much lower than that from healthy controls. Phenotypic assessment of CD80, CD83, and CD86 status showed that patients’ mo-DCs always reached a weaker maturation status compared to healthy donors’ mo-DCs, although different markers were affected according to the maturation stimulus in use. In addition to this, in vitro DC-mediated T cell stimulation was also impaired in patients, leading to subsequent lower T cell cytolytic activity and IFN-γ production [111]. Interestingly, a similar lower maturation status (lower CD80, CD83, CD86, CD40, and HLA), and functional impairment in allogenic T cell stimulation capability was also reported in mo-DCs derived from cervical cancer patients compared to healthy controls [112]. Finally, in the context of ovarian cancer, a recent study compared mo-DCs generated ex vivo from healthy subjects and ovarian cancer patients showing that, despite presenting similar levels of expression of co-stimulatory molecules, the immunogenicity of the latter was significantly impaired, as measured by MLRs [113].

In contrast with the studies reported above, Failli and colleagues, while reporting similar numerical deficiencies in levels of circulating DCs, did not observe any significant deviations from healthy donors in mo-DCs derived from melanoma patients in terms of maturation status and T-cell stimulatory capacity [76]. Instead they surprisingly noticed an increased antigen uptake in patients’ DCs compared to donors.

Therefore, despite the few discrepancies among the cited studies, which could be easily attributed to small sample size, as well as differences in disease stage, protocols, and reagents used to generate and mature mo-DCs, the evidence summarized here shows that tumors are able to negatively influence the immune fitness and immunogenicity of ex-vivo generated mo-DCs, an aspect that should be carefully considered and addressed in future DC vaccination approaches (see Section 3).

### 2.3. Other Potential Factors Implicated in Reduced Immunocompetence

It should also be underlined that most patients enrolled in clinical trials testing therapeutic cancer vaccines are elderly patients. This population of patients already have a significantly compromised immune system, due to immunosenescence [114,115,116]. Indeed, it has been proposed that immunosenescence could be an additional factor contributing to the decreased ability to control infectious disease in the elderly, as well as their generally poor response to vaccination and the increased incidence of cancer with age.

All these observations strongly support the rational of DC-based vaccines as potential anti-cancer therapies to restore functional DCs, thus promoting tumor clearance. However, on the other hand, they also point out that the immunocompromised status of cancer patients may constitute a significant barrier to this action, and concurrent further intervention should be considered to fully ensure therapeutic success.

## 3. Proposed Actions to Counteract Decreased Immunocompetent Status in Cancer Patients

Several factors could be modified when planning DC vaccination strategies to improve their clinical efficacy [117]: i) screening and assessing patients to define their immunocompetent status prior to vaccination; ii) improving vaccine formulation and defining the best route of administration (e.g., subcutaneous, intradermal, intranodal, intraperitoneal, or intravenous) to enhance efficacy; iii) optimizing vaccination schedule [118,119]; iv) and finally combining DC vaccine with different adjuvants, such as cytokines or other stimulatory factors [120]. In this section, we reviewed the current available options.

### 3.1. Patients’ Screening and Assessment

Several actions can be employed to counteract the above-mentioned cancer patients’ immune deficiencies in the context of cancer vaccine immunotherapy. First of all, a careful assessment of the patient immune status should be carried out to highlight possible chronic deficiencies, for example in the levels of circulating immune cells. As previously mentioned, the immune system deteriorates over time, and older subjects are usually characterized by decreased T-cell activity and naïve T-cell compartment [121], reduced B cell production of high-affinity antibodies [122], and, particularly important in the context of DC vaccination, a decreased number of PB-DCs in both myeloid and plasmacytoid subsets [123]. Based on these parameters, patient’s eligibility and potential responsiveness to DC vaccination should be carefully assessed before proceeding to treatment.

### 3.2. Improving DC Vaccine Formulation and Delivery Route

In recent years, the antigen source used to pulse ex vivo generated autologous mo-DCs has been recognized as a crucial function. Synthetic peptides that correspond to defined CD8^+^ epitopes have long been used in the clinic [124,125,126], however their application is only limited to patients expressing the corresponding HLA haplotypes of those epitopes. On the other hand, a more universal approach relies on autologous or allogeneic (c.f. tumor cell lines) whole tumor lysate (WTL) preparations as an antigen source [118,127]. In fact, since WTLs contain numerous specific TAAs that are characterized and un-characterized, this approach obviates the need to define specific TAAs and to select patients based on their HLA haplotypes. Furthermore, the presence of multiple epitopes restricts the chance of tumor escape by antigen loss while promoting a polyclonal CD4^+^ and CD8^+^-dependent T cell responses.

Several groups, including ours, have shown that treating tumor cells with either heat shock [128,129], hypochlorus acid (HOCl) [34,130], squaric acid [131], UV irradiation [132,133], or hydrostatic pressure [134] (among others) before DC-pulsing greatly increased their immunogenicity and improved downstream immune responses. Some of these approaches are employed in the clinical setting [135]. The enhanced tumor immunogencity could be explained in part by the induction of immunogenic cell death (ICD) [136] via these different methods mentioned above. The ICD could lead to the release of “danger signals” that activate DCs, increasing DC antigen uptake and stimulating their maturation, and ultimately improving T cell priming (see also Section 3.3.2.2). ICD might be advantageous when compared to necrotic cell death induced by more canonical free/thaw cycles [137,138]. Despite these important achievements, mechanistic insights of ICD remains unclear. A detailed elucidation of such mechanisms would further advance the DC vaccination field and potentially lead to the identification of even more immunogenic modalities.

A second central aspect in DC-based vaccines is the maturation cocktail used to fully mature DCs prior to patient’s infusion. It has been recognized that mature DCs were far superior in eliciting downstream T-cell immune responses than their immature counterparts [139,140]. This was due to the higher co-stimulatory molecules expression [141] and enhanced migratory capacity [139] of the mature DCs. The use of insufficiently matured DCs might be one of the main reasons for the failure of early DC vaccine trials [140]. Importantly, it is still unclear up-to-date what is the optimal maturation cocktail that ensures the most immunogenic DC formulation. The optimal maturation stimuli should induce high expression of MHC-I and MHC-II molecules, co-stimulatory molecules (e.g., CD40, CD80, CD86), and high secretion of Th1 inflammatory cytokines (e.g., IFN-γ, IL-12). The current standard maturation mix contains TNF-α, IL-1β, IL-6, and PGE2 [142]. Although this mix could efficiently upregulate DC surface maturation markers, it fails to induce IL-12 production [142]. Nonetheless, this cytokine cocktail is able to induce uniform DC maturation, as well as high levels of T cell proliferation and priming [142], and has thus been selected as a gold standard for maturation in many studies. Our group demonstrated that simulating WTL-pulsed DCs with LPS and IFN-γ led to strong IL-12p70 and IP-10 productions, as well as highly efficient MLRs by these activated DCs [143].

More recently, other DC maturation stimuli including CD40 ligand and IFN-γ [144], toll-like receptor ligands [145], and electroporated protein-encoding mRNA [146,147] (see Supplementary Materials Table S1) have been successfully tested. Importantly, few studies have compared the effectiveness of these different maturation cocktail on the phenotype and immunogenicity of patient-derived mo-DCs. In particular, Kvistborg and colleagues showed that mo-DCs from CRC patients matured with the gold standard mix achieved significantly higher CD83, CD86, and CCR7 than mo-DCs matured with TNF-α alone [109]. This was also observed for healthy donors. Another study used three different maturation cocktails (TNF-α/LPS, gold standard, or a mix of Ribomunyl/Imukin) to mature mo-DCs from breast cancer patients and healthy controls [111]. While the study did not specifically comment on the comparison within the cancer patient group, CD80 expression on DCs was found to be lower in the gold standard case compared to the other two. In any case, the study interestingly reported an increased IL-12 production in patients compared to the healthy controls in the case of mo-DCs matured in the presence of Ribomunyl/Imukin [111]. While these studies underlined the influence of the chosen maturation stimulus on the immunogenicity of DC vaccines, more detailed studies are clearly needed to define the optimal maturation cocktail to use.

In addition, Radice and colleagues showed that mo-DCs from non-metastatic CRC patients incubated with sequential-kinetic-activated (SKA) IL-4 and IL-12 displayed increased T cell stimulatory capacity by MLR, and an increased Th1 polarization compared to untreated mo-DCs [148]. Even if these effects were less pronounced than IL-4 and IL-12 supplemented at higher doses (ng/mL range), the authors suggest that SKA concentrations (fg/mL range) may be more clinically relevant and beneficial, leading to a more prolonged and sustained stimulation, whilst avoiding DC exhaustion [148]. Another study showed that reducing the number of injected cells improved DC migration and lymphoid homing [149], suggesting that sometimes “less is more”.

A third central aspect that needs further elucidation is the optimal route for vaccine administration. Different options have been investigated, however an optimal route has yet to emerge. Intravenous administration enables the rapid dissemination of DCs in multiple organs and lymphatic tissues, although it may lead to partial cell loss and lacks target specificity. Intranodal injection potentially constitutes an optimal route for DC delivery at their active site to promote T cell encounters, yet it is technically challenging and if improperly performed may lead to serious lymph node damage. Mouse studies have shown that intratumoral administration is able to reverse the immune suppressive TME, increasing T cell penetration and leading to tumor control [150]. However this route is not applicable to all tumors, and the TME may also negatively affect DC function and viability [151], as discussed in Section 2.1. Moreover, novel approaches based on simultaneous multiple routes of administration [6], and reported discrepancies between human and mouse models [91] have further complicated the field. Future studies aimed at correlating routes of administration with objective clinical responses would shed some light and provide future guidelines.

Finally, biomaterials such as nanoparticles have been used to deliver TAAs to naturally occurring DCs in vivo. This method has a major advantage, as it could circumvent the need to generate ex vivo mo-DCs [152].

### 3.3. Combinatorial Approaches with Additional Therapeutic Agents

#### 3.3.1. Immune Checkpoint Inhibitors

In order to maintain self-tolerance (therefore preventing autoimmunity), and to protect tissue from damage after immune activation in response to pathogens, the immune system relies on different mechanisms, collectively called “immune checkpoints”. Molecules implicated in immune checkpoints include CTLA-4 (Cytotoxic T Lymphocyte Antigen-4), PD-1 (Programmed Death-1), LAG-3 (Lymphocyte Activation Gene-3), TIM-3 (T-cell Immunoglobulin and Mucin protein-3), and several others (reviewed elsewhere; [153]). These molecules modulate T cell responses to self-proteins, as well as to chronic infections and tumor antigens. Among these molecules, CTLA-4 was the first shown to augment antitumor immune responses [154]. Following their success in melanoma and some other types of immunogenic tumors, immunomodulatory agents are currently being tested in most cancer indications, and it has been reported that therapies blocking the immune checkpoints show significant clinical efficacy in advanced tumors [155], attributed to potent activation of T-cells. Considering that lack of efficacy of DC vaccination has been associated to their inability to elicit a rapid and strong T-cell response, it is clear that combination strategies using DC vaccines with checkpoint inhibitors should generate an additive effect to overcome cancer patient’s immunosuppressive status, therefore potentially enhancing therapeutic benefit.

More specifically, this approach could be clearly advantageous for patients with gynecological and breast cancers, as most of them have been recognized as immunogenic: the host immune system can recognize and target EOC [4]; cervical cancer is mostly virally induced, and epitopes of oncoproteins E6 and E7 can be presented in the context of HLA class I molecules [156,157]; considering endometrial cancer, there is evidence suggesting that it is sufficiently immunogenic to be a reasonable candidate for immunotherapy [158]; and finally, for breast cancer (BC), the notion of immunogenic tumors is relevant for highly proliferating tumors [159], most notably for the HER2 positive and TN subtypes.

##### 3.3.1.1 Anti-CTLA-4 Treatment

The first anecdotic report indicating that anti–CTLA-4 treatment after DC vaccination might enhance DC vaccine–induced T-cell responses was published in 2005, in a dose-finding Phase I trial testing an anti-CTLA-4 monoclonal antibody [160]. There is also some evidence indicating that DC vaccination might enhance clinical efficacy of treatment with anti–CTLA-4 [161]. Additionally, DC-based immunotherapy combined with anti–CTLA-4 treatment has been shown to be more effective than the use of these agents alone in two small trials, showing a best ORR of 38% in melanoma patients [162,163]. Furthermore, in another pilot study [164], ipilimumab alone (Arm 1) or in combination with GVAX (Arm 2) was evaluated in 30 patients with previously treated, advanced pancreatic ductal adenocarcinoma (PDA) in a Phase 1b study. Of note, GVAX is a cancer vaccine composed of whole tumor cells genetically modified to secrete the immune stimulatory cytokine, granulocyte-macrophage colony-stimulating factor (GM-CSF), and then irradiated to prevent further cell division. In Arm 2, GVAX was given prior to ipilimumab to patients. Objective responses were observed in 20% of patients receiving the combination of ipilimumab and GVAX in Arm 2, whereas none of the 15 patients in Arm 1 responded to single agent ipilimumab. The median overall survival for patients in Arm 1 was 3.6 months compared to 5.7 months in Arm 2, further supporting the combination strategy. However, considering that anti-CTLA-4 monotherapy comes with higher toxicity and lower response rates than anti-PD-(L)1 (according to comparative clinical trials in melanoma patients [165,166]), increasing number of studies have focused on targeting the PD-1/PD-L1 pathway.

##### 3.3.1.2. Anti-PD-1 and Anti-PD-L1 Treatment

Initial studies with anti-PD-(L)1 were performed in patients with melanoma (a highly immunogenic tumor ), yielding impressive results [167,168], which support the current use of PD-(L)1 inhibitors as standard of care in advanced melanoma. However, immune cell interactions in the tumor microenvironment are different among tumor types, among patients, and even among tumor samples within the same patient, although some trends are commonly found. Thus, a recent systematic pan-cancer analysis has confirmed the involvement of CD8^+^ T cells in the protective anti-tumor immune response [169], in agreement with several reports over a wide range of cancer types [170,171,172,173]. In this pan-cancer analysis, infiltration from other cell types, including representative B cells, NK cells, and macrophages, was also studied: thus, infiltration from representative B cell lineage was positively associated with survival in 4 tumor types, while negatively associated with breast cancer survival; NK cell infiltration proved to be protective or showed protective trends in every cancer type examined; whereas macrophage infiltration was associated with poor patient prognosis in nine cancer types and showed trends toward negative survival associations in several other cancer types [169]. These results may explain the differential efficacy observed with anti-PD-(L)1 agents, which are currently approved by the FDA in several indications [174] (although therapy is only effective in a subset of patients for each indication), thus supporting the concept of previous patients’ assessment before immunotherapy. This is currently being implemented in the ongoing ADVISE trial (NCT03335540), as an initial clinical foray into personalized immuno-oncology therapy, aiming to analyze translational data to identify potentially actionable biomarkers across different indications (melanoma, NSCLC, RCC, UC, SCCHN, GEJ) [175].

In patients with gynecological tumors, KEYNOTE-100 (NCT02674061) showed that pembrolizumab has clinical activity in patients with advanced ovarian cancer, and PD-L1 expression (combined positive score [CPS] ≥10) was associated with response [176,177]. Recently, other Phase 1/2 trials using anti-PD-(L)1 antibodies in different combinations have shown clinical benefit in ovarian cancer: either with antiangiogenic therapy [178] (showing promising results), with poly ADP ribose polymerase (PARP) inhibitors [179] (showing responses only in a subset of patients), with pegylated liposomal doxorubicine in platinum-resistant recurrent ovarian cancer [180] (showing PFS6 = 30% [12/40 pts]), or with a folate receptor alpha (FR-α) drug conjugate [181] (showing encouraging signals of clinical activity). Similarly, clinical activity of anti-PD-1 antibodies has been also shown in patients with recurrent or advanced microsatellite instability-high (MSI-H) endometrial cancer [182], as well as in patients with recurrent or metastatic cervical cancer [183].

In triple negative BC (TNBC), ORR was 18.5% in a phase 1b study of 28 metastatic TNBC patients with pembrolizumab monotherapy [184]. In 21 metastatic TNBC bearing PD-L1-positive tumors, atelizolizumab has shown an ORR of 24% [185]. Currently, there are two ongoing studies of monotherapy with checkpoint inhibitors (KEYNOTE-086 (NCT02447003) (phase 2) and KEYNOTE-119 (NCT02555657) (randomized phase 3) [186]. There are also some ongoing studies testing anti-PD-(L)1 antibodies in combination with other therapies, such as chemotherapy: ORR of 42% has been observed in a phase IB trial of atezolizumab and nab-paclitaxel in the same setting [187], and other studies are currently ongoing, such as IMpassion130 (NCT02425891), KEYNOTE-355 (NCT02819518) (both phase 3) [186], or B-IMMUNE (NTC03356860; Phase 2, also for patients with luminal B HER2(−) tumors) [188].

Furthermore, some clinical trials are now available testing the combination of DC vaccination with anti–PD-(L)1 antibodies. For instance, in a pilot study , seven patients with metastatic pancreatic cancer received treatment with DC-based vaccine and nivolumab, given one day before the vaccine. Using response evaluation criteria in solid tumours (RECIST) , two partial responses were observed with OS after onset of therapy of 13 months and 5 months, respectively [189]. Efforts have been made to improve the anti-PD-1 antibody efficacy by extending the systemic antibody-blocking function with antibody-dependent T-cellular cytotoxicity (ADCC) properties. For example, the Fc portion of the monoclonal antibody could be kept non-mutated (e.g., avelumab anti-PD-L1) to allow it to engage the FcγRIIIa on NK cells for ADCC-mediated clearance of the large T cells [190]. Similarly, systemic blockade with anti-PD-1 [191,192] or anti-PD-L1 [193,194] in combination with DC vaccination in preclinical studies resulted in a reduction in Treg cells and an increment in the activation and activity of CTL CD8^+^ T-cells [191]. Prolonged survival was also observed in breast carcinoma [193], melanoma [194], and glioblastoma-bearing mice [192] that were treated with the combination therapy (PD-(L)1 blockade and DCs) and not with either monotherapy. Hence, the results from these preclinical studies would be useful for future clinical trial design.

Therefore, combining therapeutic vaccines with anti-PD-(L)1 antibodies (rather than anti-CTLA-4) could be safer and more feasible in the treatment of cancer. Numerous clinical trials have been initiated to test anti-PD-1 antibody in combination with DCs loaded different antigens including NY-ESO-1 (i.e., New York esophageal squamous cell carcinoma) peptides (NCT02775292), autologous whole tumor lysate (NCT03014804), and DC/tumor cell fusion vaccine against multiple myeloma (NCT01067287). Interestingly, for gynecological cancers, there is currently one Phase I trial testing the combination of Orego (an anti-CA 125 monoclonal antibody) with nivolumab (anti-PD-L1) in recurrent EOC, and further evaluation of safety and efficacy of this novel combination is ongoing in a dose expansion cohort [195]. Yet no DC vaccine in combination with anti-PD-(L)1 is currently reported in ovarian, cervical, endometrial, or breast cancer.

##### 3.3.1.3. Concomitant Blockade of CTLA-4 and PD-1/PD-L1

In preclinical models using mice with preimplanted B16 melanomas, it has been shown that concomitant blockade of both pathways can modulate Treg cell functions and enhance antitumor responses, as compared to single immune checkpoint blockade [196]. Several studies in tumor mouse models including ovarian carcinoma proved activity of the combination [196,197,198].

Consistently, nivolumab (anti-PD-L1) and ipilimumab (anti-CTLA-4) have been shown to have complementary activity in metastatic melanoma. This combination is already a standard treatment for advanced melanoma, although the combined treatment is also associated with increased toxicity: in a recent study, clinically significant immune-related adverse events (irAEs) leading to frequent emergency department visits, hospitalizations, and systemic immunosuppression were observed with 91% incidence [199].

In gynecological and breast cancers, the combination nivolumab/ipilimumab is currently being tested in one clinical trial combining different gynecological cancers (NCT03508570), as well as in other studies treating specifically breast cancer patients, either using ipi/nivo alone (NCT02892734, NCT03342417), or in combination with other therapies, such as chemotherapy (NCT03409198), cryoablation (NCT03546686, NCT02833233), or other compounds (NCT03650894, NCT02453620, NCT02983045), but not with DC vaccination. In ovarian cancer, ipi/nivo combination is also being tested alone (NCT03342417, NCT02498600, NCT03355976), as well as in endometrial cancer (NCT02982486). None of these studies use DC vaccination combined with double immune checkpoint blockade.

Hence, the efficacy of DC vaccination could be enhanced with immune checkpoint inhibitors. From a theoretical point of view, DC vaccination should be initiated first, to enhance tumor-specific immune responses, whereas subsequent immune checkpoint inhibitors should boost the initial effect, allowing induction of higher numbers of circulating T-cells.

#### 3.3.2. Other Potential Combinations to Enhance DC Vaccination

##### 3.3.2.1. Inhibiting Tumor Angiogenesis and Improving Intratumor T-cell Infiltration

Dendritic cell vaccination efficacy could also be increased when combined with therapies that break the immunosuppressive tumor microenvironment. For instance, our team recently described that the infiltration of T cells into the tumor endothelial barrier was mediated by the death mediator Fas ligand (FasL/CD95L) in the tumor vasculature of human and mouse solid tumors [200]. It was shown that FasL expression in endothelial cells was cooperatively induced by tumor-derived VEGF-A, IL-10 and PGE2, which allowed endothelial cells to selectively kill effector CD8^+^ T-cells. Yet Treg cells, which express higher levels of c-FLIP, still survived. In our study, a significant effect in tumor regression was obtained by dual inhibition of VEGF and PGE using combined anti-VEGF and aspirin. Dual VEGF and PGE inhibition lead to attenuated FasL expression, therefore allowing a marked increase in the influx of tumor-rejecting CD8^+^ over FOXP3^+^ T-cells. Hence, modulating the tumor endothelial barrier with aspirin and bevacizumab could be a promising approach to enhance antitumor responses in DC-based immunotherapy. We investigated this strategy in a pilot clinical study involving recurrent ovarian cancer patients, exploring the combination of bevacizumab, cyclophosphamide, and DC vaccine [36]. We observed a significantly higher (78%) overall survival (OS) at two years in study cohort three (vaccine plus bevacizumab/cyclophosphamide) than the corresponding 44% in the control group (a historic group of matched patients who received bevacizumab/cyclophosphamide but no DC vaccine; log-rank *P* = 0.046), the latter being similar to the reported survival for this population [201,202,203]. These observations further support the combination of DC vaccination with low-dose cyclophosphamide, which was not confirmed for bevacizumab alone.

Additionally, in several mouse tumor models it has been shown that the administration of acetylsalicylic acid (ASA) irreversibly inhibited the constitutively expressed COX1 as well as the inducible COX2. In these preclinical models, ASA combined with anti-VEGF antibody resulted in reduced tumor growth, which was associated and mediated by increased T cell infiltration [200]. Thus, PGE2 blockade in cancers can reverse the endothelial barrier and potentially synergize with T cell activation by immune-checkpoint blockade. Currently, there are a few clinical studies evaluating the effect of aspirin in combination with immunotherapy (e.g., NCT01132014).

Similarly, recent evidence in mouse models suggests that a single fraction of low-dose irradiation (LDI, i.e., 0.5-2 Gy) can reprogram the TME, inducing macrophage M1 polarization. Radiation-induced iNOS-positive M1 macrophages have been shown to produce the appropriate chemokines to recruit effector T cells, inducing tumor vasculature normalization and inflammation, therefore allowing T cell infiltration [204]. Thus, in tumors lacking T cells, LDI may be very useful as a preparatory step to induce T cell homing, previous to treatment with checkpoint inhibitors, adding minimal side effects as compared to high-dose irradiation [205].

##### 3.3.2.2. Inhibiting STAT-3 activity of tumors

This important transcription factor (STAT-3) controls tumor cell proliferation, angiogenesis, and immune tolerance, thereby constituting a bridge between oncogenesis and immunosuppression [206]. In cancer cells STAT-3 is often constitutively active [207], leading to the upregulation of VEGF and IL-10 and concomitant inhibition of DC maturation and function [206,208]. In particular, experiments in mice have shown that blocking STAT-3 activity in tumor cells abrogated the tumor-induced inhibition of DC maturation [208]. Therefore, combining STAT-3 inhibition with DC vaccines might potentially be a very fruitful approach. Several strategies and compounds are now clinically available for STAT-3 pathway blockade [209], and future efforts should be employed to combine these treatments with DC vaccination [210].

Tumor-induced accumulation of MDSCs is associated with tumor progression, immunosuppression, and immune escape [56,211], as well as affecting in particular the quality of DC vaccines [212]. Several studies have demonstrated that MDSCs elimination increased the therapeutic efficacy of DC vaccination both in mouse models [213] and in the clinic [214]. Several STAT-3 blockade strategies are available to specifically neutralize MDSCs detrimental functions, including the prevention of MDSCs generation and migration, MDSCs depletion and expansion blockade, and inhibition of MDSC immunosuppression functions [56,211,215]. Of note, MDSCs are also inhibited by STAT blockade by several drugs used for non-cancerous indications (e.g., amiloride, which is a diuretic drug used to treat high blood pressure), and natural compounds (such as icariin, the active ingredient of a herb used in Chinese medicine), having fewer side effects than anticancer drugs. These drugs could potentially be tested in the cancer setting in combination with DC vaccines.

##### 3.3.2.3. Increasing Tumor Immunogenicity with Chemotherapeutic Drugs and Radiotherapy

Interestingly, several chemotherapeutic agents have been shown to enhance the immunogenicity of tumor cells by inducing ICD, as documented by many experiments in immunocompetent mice vaccinated with tumor cells succumbing to ICD [138,216,217]. Upon exposure to chemotherapeutic agents inducing ICD, ATP release is required for the generation of an effective chemotherapy-elicited anticancer immune response [218].

In this respect, several chemotherapy agents (cyclophosphamide, oxaliplatin, or gemcitabine) have been shown to increase major histocompatibility complex (MHC) class 1 expression in tumor cells [219]. Paclitaxel, methotrexate, vincristine, and gemcitabine have a documented positive effect on DC-mediated antigen presentation [216]. In this direction, between 2001 and 2016, there have been 35 clinical trials initiated to evaluate the safety and efficacy of treatments with the combination of DC vaccines and chemotherapeutic agents activating DC directly or inducing bona fide ICD (as reported in [220]). Only limited numbers of studies have been completed and even fewer included a control group consisting of patients treated either by chemotherapeutic agents or DC-based vaccine only. However, the induction of specific antitumor immune response was observed after the administration of combinatorial therapy in most of the completed studies [221,222,223,224].

Similarly, a benefit of radiation treatment during immunotherapy has been observed, as radiation also induces immunogenic cell death [225], which has been recently reviewed elsewhere [226]. Consequently, more efforts are warranted to combine cytotoxic chemotherapy and/or radiotherapy with DC vaccines.

##### 3.3.2.4. Additional Adjuvants Specific for Therapeutic Vaccination Against Ovarian Cancer

Contrary to other solid tumors, the ovarian cancer TME is characterized by high levels of TIDCs [101,102,103]. While these DCs retain an immunogenic phenotype and activity during the early stages of cancer development, in time tumor-induced factors promote a phenotypic switch towards expansion of DCs with tolerogenic [106] and angiogenic properties [107,108], which ultimately support and accelerate malignant progression. This phenomenon is further supported by evidence in mouse models showing that early DC depletion is detrimental towards disease control, while depletion in later or metastatic stages strongly inhibits tumor growth [106]. Hence, it has been proposed that dendritic cell depletion performed in combination to administration of DC vaccines in the context of advanced ovarian cancer can potentially improve therapeutic otucomes [108]. Notably, this is also the context were most therapeutic intervention is done, due to the rather asymptomatic features of early stage ovarian cancer and hence rare diagnoses [227,228]. In alternative, thanks to their highly occurrence in the ovarian TME, local reprogramming and re-activation of TIDCs towards an immunogenic phenotype may also hold important therapeutical benefits. To this aim, through the administration of siRNA encapsulating nanoparticles, Cubillos–Ruiz and colleagues were able to directly induce a TIDC phenotypic switch from tolerogenic to immunogenic phenotype, leading to T cell activation, tumor shrinkage, and increased animal survival in an ovarian cancer mouse model [229]. A similar in situ reprogramming of ovarian cancer TIDCs was also reported by Scarlett and colleagues through CD40 and TLR3 stimulation [230]. Importantly, in addition to the reported beneficial effects, this approach presents also the further advange of overcoming the need for generating moDCs ex vivo, an expensive and cumbersome process, and is therefore worthy of further efforts towards its clinical translation. Finally, while previous reports demonstrated the presence of an immunosuppressive TME also in other gynecological cancers such as cervical [231] and uterine/endometrial cancers [158], the occurence and contribution of TIDCs has not been investigated so far, at least to our knowledge. Future work should be carried out to address this point and evaluate the applicability of the above mentioned therapeutic approaches to re-educate the immunosuppressive and pro-tumor TME towards an immunogenic one.

### 3.4. Potential Supporting Actions that Could Add Therapeutic Benefit

As previously indicated (refer to Section 2.3) immunosenescence in elderly cancer patients can be another factor hampering vaccination efficacy. Therefore, additional interventions might be required in elderly patients to boost T cell immunity. Although different options are currently under investigation [232], some of the proposal are: i) provide IL-7 (a T cell survival factor) as an immune rejuvenating agent, which has been explored in the mouse [233]; ii) modify the rate of thymic involution by therapeutic modulation of the neuroimmunoendocrine axis [234]; iii) transfusion of autologous leukocytes after prolonged storage [235]; iv) nutritional interventions aiming to improve T cell function in elderly patients, such as supplemented diet with an energy source and trace elements [236], with vitamin E [237], or conjugated linoleic acid [238], as well as controlling cholesterol levels [239].

## 4. Discussion

Dendritic cell vaccination is currently considered an interesting immunotherapeutic option, due to low immune-related toxicity [8,9], and preserved quality of life of cancer patients [14]. The observed low clinical benefit form DC vaccines is probably associated with a deficicient immunocompetent status in cancer patients, translated as reduced capacity to activate antitumoral T cells. The potential factors that could negatively affect DC vaccination are summarized below, together with proposed counteracting factors.

According to current data, we can conclude that tumor cells, partly by releasing specific cell growth and signalling factors, are able to induce immune system deficiencies, in particular DC defects promoting tumor immune escape and disease progression, such as a decreased total number of DCs circulating in the peripheral blood (PB-DCs) [76,79,80,81,84], with an observed reverse trend towards normalization in terms of DC numbers before and after tumor surgical removal [80,81,86,87,88], indicating that these numerical deficiencies were tumor-induced, and reversible upon debulking procedures. Additionally, important DC functional impairments have also been reported in several types of cancer [82,87,88,89], as compared to healthy subjects. The rational of DC-based vaccines as potential anti-cancer therapies to restore functional DCs in order to enhance tumor clearance are thus strongly supported by these observations. Additionally, important defects have been reported in monocyte-derived DCs (mo-DCs) cultured ex vivo for therapeutic vaccination, as compared to mo-DCs derived from healthy subjects [76,109,110,111,112]. This observation suggests that the immunocompetence status of responders was positively influenced by the therapeutic effects of DC vaccination. Therefore, as the immunocompromised status of cancer patients may constitute a significant barrier to vaccination, concurrent further intervention should be considered to enhance therapeutic success. These observations are generally applicable across all cancer types, as well as specifically to gynecological and breast cancers.

Firstly, patient eligibility and potential responsiveness to DC vaccination should be carefully assessed before proceeding to treatment. Additionally, selecting patients with either minimal burden disease, or after debulking strategies (whenever possible) may significantly improve the efficacy of DC vaccination. Another factor contributing to the reduced efficacy of therapeutic vaccination is immunosenescence [114,115,116], as most patients enrolled in clinical trials involving therapeutic cancer vaccines are elderly patients, requiring additional interventions to boost T cell immunity [232], including both interventional [233,234,235], or nutritional measures [236,237,238,239].

Considering potential actions on DC vaccines themselves, in recent years WTLs have been recognized as an excellent antigen source to pulse ex vivo generated autologous mo-DCs, as they present several advantages. The immunogenicity of this approach has been further increased due to recent developments in the context of WTL vaccination. The identification of even more immunogenic modalities in the future could lead to a detailed elucidation of such mechanisms, allowing the selection of one or few among them. We can also conclude that several parameters including antigen source, maturation stimulus, and route of administration play a crucial role in the overall success of DC-based cancer vaccines. Future careful and systematic comparison studies should clarify which conditions are optimal and most immunogenic to achieve the besT-cellular product, although several advancements have been recently made in these areas.

Finally, it is important to note that combinatorial approaches using DC vaccines may significantly increase efficacy, taking advantage of their positive safety profile. Several different options are currently under investigation, using different agents in combination with DC vaccines, aiming to trigger concurrent T cell activation (using checkpoint inhibitors), break immunosuppression (using agents such as anti-VEGF, COX inhibition, STAT-blockade, or LDI), and/or increase immunogenicity (using standard chemotherapy agents, or radiotherapy). Future results from ongoing studies should indicate the most appropriate combination in each indication.

## Supplementary Materials

The following are available online at http://www.mdpi.com/2076-393X/6/4/79/s1, Table S1: List of published studies with DC vaccines in gynecological and breast cancers, from 2000 to date.
