# Supplementary material for: Does the Immunocompetent Status of Cancer Patients Have an Impact on Therapeutic DC Vaccination Strategies?
Source: Vaccines (Basel). 2018 Nov 23;6(4):79. doi: 10.3390/vaccines6040079 (PMC6313858; doi:10.3390/vaccines6040079)
Supplement: Supplementary file 1 [file vaccines-06-00079-s001.pdf]

Supplementary Materials

Table S1. List of published studies with DC vaccines in gynecological and breast cancers, from 2000 to date.

Reference / Study type	Study indication	Vaccine type and preparation	DC Matur. stimulus	N° of pts	Results: immunogenicity	Results: survival (PFS/OS)	AEs
**Brossart-2000 [21]/ Phase I**	Metastatic BrCa or OvCa that expressed HLA-A2 and HER-2/neu or MUC1.	* DC peptide vaccine: Her2/neu, MUC1.<br>* Peptide-pulsed DCs generated from PBMCs were injected s.c. into the upper limb close to the inguinal lymph nodes on days 1, 14, and 28, respectively.<br>* PBMCs isolated from blood, were cultured in medium supplemented with IL-4, GM-CSF, and TNF-α. DCs were separately pulsed for 2 hours with each peptide, and washed before application.<br>* Peptides derived from HER-2/neu (E75: KIFGSLAFL, GP2:	TNF-α	10 (7 BrCa, 3 OvCa)	* In 5 of 10 vaccinated pts, peptide-specific CD8+ cytolytic T-cells were detected in the peripheral blood after 3 Vx<br>* Epitope spreading with a single tumor antigen was observed in vivo on vaccination	No data	* None, particularly no clinically relevant anemia.<br>* No autoimmune phenomena observed

		IISAVVGIL) and MUC1 (M1.1 STPPVHNV, M1.2: LLLLTVLTV) were synthesized<br>* Pts received either HER-2/neu or MUC1 peptides					
**Triozzi-2000 [20]/ Phase I**	Pts with metastatic dermal or subcutaneous tumors (BrCa, MEL)	* DC vaccine<br>* DCs generated from monocytes obtained by phlebotomy and cultured with GM-CSF and IL-4 in autologous plasma for 8 days<br>* Tumors were injected at multiple sites with 30 million autologous DCs per tumor	no maturation	10 (3 BrCa, 7 MEL)	* Biopsies of regressing lesions showed lymphocyte infiltration (TIL) associated with DCs and necrosis<br>* Injected DCs produced IFN-a and expressed Fas ligand mRNA<br>* No cytolytic activity in vitro (reduced expression of the costimulatory molecule, B7-2 (CD86) on DCs after IT injection)	* 4 days after injection, regression of the injected tumors was observed in 4 MEL pts and in 2 BrCa pts	* Injections were well tolerated.<br>* Only 1 pt reported pain (<48h)

**Hernando-2002 [31]/ Phase I**	Progressive or recurrent OvCa or uterine sarcoma.	* DC aWTL and KLH vaccine.<br>* DCs pulsed with autologous tumor lysate and KLH.<br>* Crude lysates from tumor specimens (WTL) were frozen.<br>* PBMCs isolated from leukapheresis were cultured for 6-7 days with GM-CSF.<br>* Inmature DCs were pulsed with KLH and autologous WTL in the presence of GM-CSF, IL-4 and TNF-α<br>* Cells harvested in day 7 or 10 were administered i.c. in close proximity to the axillary lymph nodes.<br>* pts received 3 to 23 injections of KLH- and WTL-pulsed DC Q10D or Q4W	TNF-α	8 (6 OvCa, 2 UtSar)	* A significant tumor antigen-specific lymphoproliferative response was detected in 2 pts after two Vx	* A possible correlation between the immune response and disease stabilization was suggested	* Treatment was safe, feasible and well tolerated<br>* No major toxic (grade 2) or SAE related to the vaccine<br>* Minor general effects included mild transient fatigue, chills and low-grade fever (2 pts); no treatment required<br>* No rash or lymphadenop athy or autoimmunity

**Vonderheide-2004 [22]/ Phase I**	HLA-A2-positive pts with hormone-independent prostate cancer or refractory progressive metastatic BrCa.	* DC peptide hTERT vaccine: autologous DCs loaded with hTERT peptide and KLH<br>* Generated from PBMCs<br>* Matured with GM-CSF and IL-4, and pulsed on day 7 with KLH and one of three peptides (hTERT I540, HIV RT-pol476 or influenza MP58)<br>* Eligible pts were s.c. administered a total of 15 × 10^6 autologous DCs every other week for up to six Vx	no maturation	7 (5 ProstCa, 2 BrCa)	* hTERT-specific T lymphocytes induced in 4 of 7 pts<br>* hTERT-specific CD8+ cells after vaccination were identified by peptide/MHC tetramers, proliferated, and secreted IFN-γ after in vitro peptide sensitization, killed tumors, and demonstrated phenotypic characteristics of tumor-lytic CD8+ T-cells	* PR in 1 pt was associated with the induction of CD8+ TILs	* No significant toxicity observed
**Svane-2004 [23]/ Phase I**	HLA-A2+ pts with progressive advanced BrCa.	* DC peptide p53 vaccine: autologous dendritic cells (DCs) loaded with 3 wild-type and 3 P2 anchor modified HLA-A2 binding p53 peptides (designed to increase HLA-A*0201 binding	no maturation	6	* Specific T-cell responses against modified and unmodified p53 peptides observed in 3 pts, including 2 of the pts with a possible clinical benefit from the treatment.	* SD was seen in 2 of 6 pts, 1 pt had a transient regression of a single lymph node, and 1 had a mixed response.	* Vx were well tolerated<br>* No irritation at the site of injection; no allergic or autoimmune reactions; no haematologic,

		capacity and induction of p53-specific cytotoxic T lymphocytes)<br>* PBMCs cultured for 7 days with IL-4 and GM-CSF and then frozen<br>* Cells pulsed with six HLA-A2-associated p53 peptides, and a pan-MHC class II peptide, PADRE, for 2 h at 37C.<br>* Pts received up to 10 s.c. Vx with 5×10^6 p53-peptide loaded DC with 1–2 weeks interval. Concomitantly, IL-2 was administered s.c.					hepatic, pulmonary or renal toxicities<br>* Most common side effect: mild to moderate local reaction at the site of Proleukin injection. All pts experienced mild flu-like symptoms lasting 12–24 h after injection of Proleukin.
**Avigan-2004 [40]/ Phase I**	Pts with metastatic BrCa and renal cancer (RC), with tumor lesions accessible to biopsy or resection	* DC vaccine: prepared by fusing autologous tumor cells and DCs<br>* Tumor tissue was disrupted into single cell suspensions.<br>* PBMCs by leukapheresis were cultured in GM-CSF, IL-	no maturation	23 (10 BrCa, 13 RC)	* Fusion cells coexpressed tumor and DC antigens and stimulated allogeneic T-cell proliferation.<br>* In a subset of pts, an increased percentage of CD4 and CD8+ T-cells expressing intracellular IFN-gamma in response to in	* 2 pts with breast cancer showed disease regressions, including a near complete response of a large chest wall mass<br>* SD in 5 pts with RC and 1 pt with BrCa	* No significant treatment-related toxicity<br>* No clinical evidence of autoimmunity

		4, and autologous plasma. Tumor cells and DCs were cocultured with PEG to generate the fusions. * Fusion cells were administered s.c. Q3W			vitro exposure to tumor lysate was observed		
**Danet-Desnoyers -2005 [24]/ Phase I**	HLA-A2-positive pts with hormone-independent prostate cancer or refractory progressive metastatic BrCa.	* DC vaccine: autologous DCs loaded with hTERT peptide and KLH * From PBMCs. DCs matured with GM-CSF and IL-4, and pulsed on day 7 with KLH and one of three peptides (hTERT I540, HIV RT-pol476 or influenza MP58). * Eligible pts were s.c. administered a total of 15×10^6 autologous DCs every other week for up to six Vx.	no maturation	5	* No significant decline in the frequency of granulocyte, macrophage or erythroid CFCs using CFC assays or long-term in vitro cultures * In NOD/SCID mice, human hematopoietic reconstitution was easily detected, without quantitative or qualitative differences between pre- and postvaccine samples	See Vonderheide, 2004	See Vonderheide, 2004

**Svane-2007 [25]/ Phase II**	HLA-A2+ pts with progressive advanced BrCa.	* DC vaccine: autologous DCs loaded with 3 wild-type and 3 P2 anchor modified HLA-A2 binding p53 peptides (designed to increase HLA-A*0201 binding capacity and induction of p53-specific cytotoxic T lymphocytes)<br>* PBMCs cultured for 7 days with IL-4 and GM-CSF and frozen<br>* Cells pulsed with six HLA-A2-associated p53 peptides, and a pan-MHC class II peptide, PADRE, for 2 h at 37°C<br>* Pts received up to 10 s.c. Vx with 5×10^6 p53-peptide loaded DC with 1–2 weeks interval. Concomitantly, IL-2 was administered s.c.	no maturation	26	* Therapy-induced p53 specific T-cells were observed in 4/7 pts with SD but only in 2/9 pts with PD.<br><br>* See Svane, 2008	* 19 pts available for first evaluation after 6 Vx<br>* SD or minor regression observed in 8/19 evaluable pts or minor regression<br>* PD in 11/19 pts	* Vaccine was well tolerated.<br>* No skin toxicity at the site of vaccine injection<br>* No autoimmunity<br>* Most common side effect: mild to moderate local reaction at the site of proleukine injection<br>* Pts experienced CTC grade 1–2 flu-like symptoms 12–24 h after proleukine injection

**Svane-2008 [26]/ Phase II**	HLA-A2+ pts with progressive advanced BrCa.	* DC vaccine: autologous DCs loaded with 3 wild-type and 3 P2 anchor modified HLA-A2 binding p53 peptides (designed to increase HLA-A*0201 binding capacity and induction of p53-specific cytotoxic T lymphocytes).<br>* PBMCs cultured for 7 days with IL-4 and GM-CSF and frozen.<br>* Cells pulsed with six HLA-A2-associated p53 peptides, and a pan-MHC class II peptide, PADRE, for 2 h at 37°C.<br>* Pts received up to 10 s.c. Vx with 5×10^6 p53-peptide loaded DC with 1–2 weeks interval. Concomitantly, IL-2 was administered s.c.	no maturation	26	* Any significant differences between SD and PD pts<br>* Decrease in naïve T-cells during vaccination, in particular in pts with PD.<br>* The frequency of CD4+ CD25high T-cells was almost doubled after only four weeks of weekly vaccination and IL-2 dosing.<br>* More than 90% of the CD4+ CD25 high T-cells co-expressed foxp3 confirming the regulatory functionality of these T-cells, independent of clinical response	See Svane, 2007	See Svane, 2007

**Santin-2008 [29]/ Phase I**	pts with stage IB or IIA cervical cancer (non parametrial involvement), tumor HPV 16/18+.	* THER; DC protein HPV16/18 E7/KLH vaccine.<br>* PBMCs cultured with GM-CSF<br>* At day 4, DCs were pulsed overnight (12 to 16 h) with HPV16/18 E7 proteins and KLH at a dose of 50 μg/ml<br>* At day 5, maturation was induced by 48h incubation with TNF-a, IL-1b and PG-E2a<br>* HPV16/18 E7/KLH-pulsed DC were injected s.c. 10 cm inferior to the inguinal ligament of the anterior mid-thigh.<br>* Five DC Vx were performed Q21D	IL-1β, PGE2, TNF-α	10	* All pts developed CD4(+) T-cell and antibody responses to DC vaccination<br>* 8 out of 10 pts demonstrated levels of E7-specific CD8(+) T-cell counts<br>* Vaccine dose did not predict the magnitude of the antibody or T-cell response or the time to detection of HPV16/18 E7-specific immunity.<br>* DTH responses to intradermal injections of HPV E7 antigen and KLH were detected for all pts after vaccination.	* No sign of tumor recurrence detected in any of the treated pts up to the time of writing	* Well tolerated, no significant toxicities recorded<br>* No significant local or systemic reactions<br>* No allergic reacions<br>* No alteration detected in liver and renal function<br>* Local reactions detected (mild erythema, swelling/ induration, and pruritus) at the s.c. vaccination sites

**Wang-2009 [30]/ Retrospective**	pts with stage IB or IIA cervical cancer (non parametrial involvement), tumor HPV 16/18+.	* THER; DC protein HPV16/18 E7/KLH vaccine.<br>* PBMCs cultured with GM-CSF<br>* At day 4, DCs were pulsed overnight (12 to 16 h) with HPV16/18 E7 proteins and KLH at a dose of 50 μg/ml<br>* At day 5, maturation was induced by 48h incubation with TNF-a, IL-1b and PG-E2a<br>* HPV16/18 E7/KLH-pulsed DC were injected s.c. 10 cm inferior to the inguinal ligament of the anterior mid-thigh.<br>* Five DC Vx were performed Q21D	IL-1β, PGE2, TNF-α	8	* 12 T-cell lines from 8 subjects (7 HPV 16-positive, 1 HPV 18-positive) evaluated<br>* Positive T-cell responses in 4 subjects (all HPV 16-positive), all positive for the HPV 16 E7 46-70 (EPDRAHYNIVTFCCKCDSTLRLCVQ) region<br>* T-cell clones specific for the E7 47-70 region were isolated from 1 subject<br>* Further analyses revealed a novel, naturally processed, CD4 T-cell epitope, E7 58-68 (CCKCDSTLRLC), restricted by the HLA-DR17 molecule	See Santin, 2008.	See Santin, 2008.
**Baek-2011 [32]/ Phase I/II**	Advanced RCC or BrCa.	* DC vaccine: DCs pulsed with autologous tumor lysate (WTL) and KLH.<br>* Cancer pts were	IFN-γ	10 (6 RCC, 4 BrCa)	* Peripheral blood lymphocyte proliferation and the number of IFN-r secreting cells were induced in 6 pts without clear correlation with clinical	* Clinical response was observed in one RCC pt as SD<br>* 9 cases showed PD	* Well tolerated without major side effects

		treated twice with autologous CD34+ hematopoietic stem cell-derived, GM-CSF/IFN-γ-differentiated DCs (cultured during 7+7 days), pulsed with autologous WTL and KLH, Q4W.<br>* Following each s.c injection of therapeutic DCs, low-dose (200 MIU) IL-2 was introduced for 14 consecutive days			responses.<br>* NK activity was induced significantly in 6 pts after vaccination.<br>* DC vaccine-related decrease of TGF-β level or increase of IL-12p70 level and decline of CD4+CD25+ T-cells were observed in 3 pts		
**Chu-2012 [27]/ Phase I/II**	HLA-A2-positive pts with advanced epithelial OvCa or primary peritoneal cancer in remission.	* DC vaccine: hTERT, Her2/neu, PADRE.<br>* Monocyte-derived DCs loaded with synthetic peptides, with or without low-dose intravenous Cy. All pts also received pneumococcal vaccine.<br>* PMBCs by leukapheresis cultured in the presence of GM-	membrane fractions from *Klebsiella pneumoniae* and IFN-γ	14 OvCa	* Pts receiving Cy had a transient reduction in neutrophils, but no change in total lymphocytes or regulatory T-cells<br>* Modest T-cell responses to Her2/neu and hTERT were seen post-vaccine by IFN-γ ELISPOT<br>* Pts demonstrated below normal responses to the diphtheria conjugate protein	* 3-year OS 90% (no difference for pts receiving Cy over controls)<br>* Estimated 3-year PFS was 40% versus 80% for Arms 1 (without Cy) and 2 (with cy), respectively; the estimated 3-year OS was 80 and 100%,	* No grade 3/4 vaccine-related toxicities were noted<br>* Most common study-related toxicities: reactogenicity as indicated by erythema,

		CSF and IL-13 for 7 days to generate DCs<br>* Maturation by culture with membrane fractions from Klebsiella pneumoniae and IFN-γ<br>* Mature DCs were pulsed with HLA-A2-restricted hTERT 988Y, Her2/ neu 369V2V9, Her2/neu 689, and PADRE peptides and cryopreserved<br>* Each dose was injected into the medial thighs at 24 intradermal sites			CRM197, a component of the pneumococcal vaccine	respectively<br>* Of 11 pts, 2 recurred during vaccination. Nine received all 4 doses: 3 pts recurred at 6, 17, and 26 months, respectively, and 6 have no evidence of disease at 36 months.	induration, pruritus, and pain at the site of injection, fever and fatigue
**Qi-2012 [33]/ Feasibility**	Double-negative stage II/IIIA BrCa.	* DC vaccine: autologous dendritic cells pulsed with autologous tumor lysates (WTL)<br>* DC vaccines generated from CD14+ precursors pulsed with autologous WTL<br>* DCs were matured GM-CSF and IL-4	IL-1β, PGE2, TNF-α	31	* DC vaccines elicited Th1 cytokine secretion and increased NK cells, CD8+ IFN-+ cells but decreased the percentage of CD3+ T-cells and CD3+ HLA-DR+ T-cells in the peripheral blood<br>* Approximately 58% (18/31) of pts had a DTH-positive reaction	* No difference in OS between the pts with and without DC vaccine<br>* 3-year PFS was significantly prolonged: 76.9% versus 31.0% (with vs. without DC vaccine, $p < 0.05$).	* No unanticipated or SAEs<br>* A self-limited wheal-and-flare skin reaction appeared at the injection site 24–48 h after

		* Tumor antigens added on day 5. On day 6, Il-1β, PGE2 and TNF-α added<br>* Cells harvested on day 7 and directly used. Individuals were immunized intradermally 4 times					immunization in 20 of 31 subjects during at least one of the four immunizations<br>* No abnormalities in hematological parameters or serum chemistries
**Chiang-2013 [34]/ Pilot**	Recurrent OvCa.	* DC vaccine: OCDC<br>* DC vaccine pulsed with HOCl-oxidized tumor lysate (WTL; see OvCa-Vac-Kandalaft-2013).<br>* OCDC were administered through direct injection into the one to two groin lymph nodes bilaterally under ultrasound guidance	LPS, IFN-γ	5 OvCa	* Subjects' DCs behaved similar to normal donor DCs, producing high levels of IL-12 and other important Th1 cytokines and chemokines<br>* Following vaccination, a potent systemic inflammatory activation was confirmed in these subjects<br>* Tumor-reactive T-cells exhibited a strong Th1 polarization<br>* OCDC vaccine was highly	* 2 subjects remained in remission for a period of time much longer than expected based on historic observations<br>* Both subjects demonstrated a PFS2 > PFS1 in response to OCDC vaccination as second-line therapy following relapse	* All vaccines well tolerated; most toxicities were <grade 2<br>* Common side effect: flu-like symptomatology (i.e. fatigue, fever and chills)

		* All subjects completed 5 Vx (Figure 4A), except S1 who withdrew after 3 Vx due to disease progression.			efficient in crosspresentation to CD8+ T-cells. This vaccine also efficiently primed tumor-specific CD4+ T-cell response.	from first-line therapy	
**Kandalaft-2013 [35]/ Phase I**	Relapsed epithelial carcinoma arising in the ovary, fallopian tube, or peritoneum	* DC vaccine: OCDC.<br>* DC vaccine pulsed with autologous oxidized WTL, in combination with antiangiogenesis therapy (bevacizumab) and metronomic Cy (three-arms study)<br>* Faster, four-day protocol for DC preparation, using GM-CSF, IL-4 and serum-free AIM-V media	LPS, IFN-γ	25 OvCa	* Day-4 DCs generated with this protocol are similar to "classic" Day-7 DCs, in terms of phenotype and phagocytic capability, and have a higher capacity than Day-7 DCs to produce IL-12p70 following LPS and IFN-γ stimulation<br>* In addition, these Day-4 DCs were highly immunogenic	No data	No data
**Kandalaft2-2013 [36]/ Pilot**	Recurrent OvCa pts for whom tumor lysate was available from prior cytoreductive surgery.	* DC vaccine: OCDC.<br>* DC vaccine pulsed with autologous oxidized WTL, in combination with antiangiogenesis therapy (bevacizumab) and metronomic Cy	LPS, IFN-γ	6	* All subjects exhibited a dampened T-cell response to the diphtheria carrier protein CRM197, given along with the first Vx to monitor immune responsiveness<br>* DC Vx induced an immune response against WTL and	* 4 of 6 pts (66%) achieved clinical benefits with the combination of bevacizumab, metronomic Cy and the vaccine (2 PR and 2 SD)	* All vaccines well tolerated and no grade > 2 toxicities.<br>* Most frequent AE: grade 1 or 2 hypertension

	(three-arms study)<br>* PBMCs cultured for 6 days with IL-4 and GM-CSF. Immature DCs incubated for 18h with the oWTL, IL-4 and GM-CSF<br>* Pts underwent conditioning with intravenous bevacizumab and oral metronomic Cy, sequentially followed by (1) bevacizumab plus vaccination with DCs pulsed with autologous tumor cell lysate supernatants, (2) lymphodepletion and (3) transfer of 5×10^9 autologous vaccine-primed T-cells in combination with the vaccine		specific immune responses against peptides of known tumor-associated antigens such as HER2<br>* Increased IgM seropositivity post-vaccine was detected<br>* Frequency of tumor-specific T-cells elicited by vaccine quite low (< 1 tumor reactive T-cell per 500 PBLs)	* 1 subject: post-vaccine remision of 14 mo in spite of a prior PFS of 7 mo	(3 occurrences), attributed to bevacizumab

**Kobayashi -2014 [28]/ Pilot**	Recurrent OvCa, any HLA type.	* DC vaccine: WT1, MUC1, CA125.<br>* Each pt was first evaluated for human leukocyte antigen (HLA) expression, to determine the type of peptides for administration<br>* PBMCs from leukapheresis cultured for 5 days in medium with GM-CSF and IL-4 to generate immature DCs<br>* Maturation with OK-432 and PG-E2 for 24h, then pulsed with the selected peptides, and cryopreserved<br>* All pts were i.d. injected 5–7 times with DCs (approximately 107 cells/injection) in close proximity to the axial and/or inguinal lymph nodes. Injections were	OK-432, PG-E2	56 OvCa	* No remarkable changes observed in CD4+ T-cell, CD8+ T-cell, and NK cell frequencies after vaccination; in addition, none of these factors affected the median survival time (MST)<br>* No significant differences in clinical outcomes between pts with WT1-specific CTL increase and those without such an increase (17 pts evaluable)	* Clinical response evaluated in 56 pts at 3 and 6 months. The MST from diagnosis was 30.4 months and that from the first vaccination was 14.5 months.<br>* The 1- and 2-y OS from diagnosis were 87% and 65%, resp. At the time of the final analysis, 35 pts (63%) had died of cancer.<br>* At 3 mo after the first Vx, any pt showed CR. However, 2 pts (3.6%) had PR, 14 (25%) SD, 32 (57%) PD, and 8 (14%) were not evaluated. The DCR and ORR were 29% and 3.6%, resp.	* Tolerable in all pts<br>* Most common: injection site reaction (68%) and fever (32%)<br>* No serious acute allergic reaction such as anaphylaxis, or other common AEs such as arthralgia and elevated liver enzyme levels<br>* No grade 3–4 toxicity or evidence of autoimmune sequelae

		repeated every 14–21 days<br>* OK-432, a streptococcal immunological adjuvant, was administered simultaneously with the DC vaccine to pts without serious allergies to penicillin or other drugs					
**Ramanathan-2014 [37]/ Phase I**	Advanced, recurrent cervical cancer pts who had failed conventional therapy, HPV+.	* THER; DC WTL vaccine.<br>* PBMCs were cultured for 7 days with GM-CSF and IL-4. Antigen loading was evaluated using three strategies: tumor lysate, cervical cancer cell line lysate (from HeLa, SiHa and C33A cells) and tumor RNA. Tumor lysate was added to immature DC.<br>* After 4 h of exposure to antigen, IL-1β and	IL-1β, TNF-α	14	* One pt showed improved proliferation of lymphocytes after the third vaccination but the increase however was not significant (paired two tailed t test =0.06)<br>* No significant proliferation responses were seen in any of the other pts<br>* Comparing arm II and arm III pts' responses also did not show significant difference	* One pt who received WTL-primed DCs and later cis-platin chemotherapy showed a CR of her large metastatic disease and remained disease free for more than 72 months	* According to WHO criteria, grade 0 or grade one toxicity was observed in three pts<br>* None of the 9 pts evaluated had any elevation of auto antibody levels after the third vaccination

		TNF-α were added and the cells were incubated for 3 days. On day 10, the mature DC were frozen.<br>* Vx were given to each pt intra-dermally. Pts received three Vx, one every 14 days. A maximum of 1x10^6 cells were given at each vaccination				although 1 pt, who was in arm I (saline only) showed slight elevation of ANA levels
**Baek-2015 [38]/ Phase I/II**	OvCa pts with minimal residual disease	* DC aWTL vaccine: DCs pulsed with autologous tumor lysate and KLH.<br>* Cancer pts received GM-CSF (injected s.c.) during 5 days, for monocyte mobilization, and PBMCs were then collected by leukapheresis. Cells were cultured with CM-CSF and IL-4 for 7 days. On day 7, TNF-a was added for 2 days	TNF-α	10	* In the 3 pts with disease free long-term survival, significant immune alterations were observed, including increased natural killer (NK) activity, IFN-γ-secreting T-cells, immune-stimulatory cytokine secretion and reduced immune-suppressive factor secretion after DC vaccination	* In 3 out of 10 pts, the inclusion status after the initial therapy showed the maintenance of CR after DC vaccination for 83, 80.9 and 38.2 months without disease relapse<br>* 1 pt with SD experienced the complete disappearance of tumor after DC vaccination,	The vaccination was well tolerated. The most common side effects were flu-like symptoms.

		* DCs were pulsed with aWTL and KLH on day 8 O/N. On day 9, cells were harvested, washed, resuspended and tested<br>* Three to seven months after the initial surgery and chemotherapy, 10 pts were treated with aWTL-loaded DCs ($4.13\times10^7\pm0.27\times10^7$ cells/injection), followed by 14 consecutive IL-2 (200 mIU) injections in a single vaccination protocol<br>* Two Vx were s.c. administered in an area adjacent to the axillary lymph node Q4W				maintained for 50.8 months until tumor recurrence<br>* In 2 pts with PR was not responding to DC vaccination and their disease recurred	
**Tanyi-2018 [39]/ Pilot**	platinum-treated, immunotherapy-naïve, recurrent OvCa pts	* Pilot clinical trial testing a personalized vaccine (OCDC) generated by autologous DCs pulsed with oxidized	LPS, IFN-γ	6	* Vx induced T-cell responses to autologous tumor antigen associated with significantly prolonged survival<br>* Vx also amplified T-cell responses against mutated		* A total of 392 vaccine doses were administered without

	autologous whole-tumor cell lysate (aWTL), which was injected intranodally in platinum-treated, immunotherapy-naïve, recurrent ovarian cancer pts	neoepitopes derived from nonsynonymous somatic tumor mutations, including priming of T-cells against previously unrecognized neoepitopes, as well as novel T-cell clones of markedly higher avidity against previously recognized neoepitopes	serious adverse events

Abbreviations: aWTL, autologous whole tumor lysate; BrCa, breast cancer; CR, complete response; Cy, cyclophosphamide; DC(s), dendritic cell(s)s; i.d., intradermal; i.v., intravenous; Matur, maturation; MEL, melanoma; OS: overall survival; OvCa, ovarian cancer; PFS, progression-free survival; ProstCa, prostate cancer; PR, partial response; Pt(s), patient(s); RC, renal cell carcinoma; resp., respectively; s.c., subcutaneous; SD, stable disease; UtSar, uterine sarcoma; Vx, vaccinations.
